# Dynamics of the Developing Chick Chorioallantoic Membrane Assessed by Stereology, Allometry, Immunohistochemistry and Molecular Analysis

**DOI:** 10.1371/journal.pone.0152821

**Published:** 2016-04-05

**Authors:** Andrew Ndegwa Makanya, Ivanka Dimova, Tobias Koller, Beata Styp-Rekowska, Valentin Djonov

**Affiliations:** 1 Department of Veterinary Anatomy and Physiology, Riverside Drive, Chiromo Campus, University of Nairobi, Box 30197, 00100, Nairobi, Kenya; 2 Department of Medical Genetics, Medical University Sofia, Zdrave street 2, 1431, Sofia, Bulgaria; 3 Institute of Anatomy, University of Bern, Baltzerstrasse 2 CH-3000, Berne, 9, Switzerland; University of Bari Medical School, ITALY

## Abstract

The chick chorioallantoic membrane (CAM) is a widely used model for the study of angiogenesis, tumour growth, as well as drug efficacy. In spite of this, little is known about the developmental alteration from its appearance to the time of hatching. In the current study the CAM has been studied by classical stereology and allometry. Expression levels of selected angiogenesis-related molecules were estimated by RT-PCR and cell dynamics assessed by proliferation and apoptosis assays. Absolute CAM volume increased from a low of 0.47 ± 0.11 cm^3^ at embryonic day 8 (E8) to a high of 2.05 ± 0.27 cm^3^ at E18, and then decreased to 1.6 ± 0.47 cm^3^ at E20. On allometric analysis, three growth phases were identifiable. Between E8-13 (phase I), the CAM grew fastest; moderately in phase II (E13-18) but was regressing in phase III (E18-20). The chorion, the mesenchyme and the allantoic layers grew fastest in phase I, but moderately in phase II. The mesenchyme grew slowly in phase III while the chorion and allantois were regressing. Chorionic cell volume increased fastest in phase I and was regressing in phase III. Chorionic capillaries grew steadily in phase I and II but regressed in phase III. Both the chorion and the allantois grew by intrinsic cell proliferation as well as recruitment of cells from the mesenchyme. Cell proliferation was prominent in the allantois and chorion early during development, declined after E17 and apoptosis started mainly in the chorion from E14. VEGFR2 expression peaked at E11 and declined steadily towards E20, VEGF peaked at E13 and E20 while HIF 1α had a peak at E11 and E20. Studies targeting CAM growth and angiogenesis need to take these growth phases into consideration

## Introduction

The chorioallantoic membrane (chorioallantois, CAM) is a vascular membrane found in embryonated eggs of some amniotes, such as birds and reptiles. It results from the fusion of the mesodermal layers of two developmental structures: the allantois and the chorion [1]. In mammals, the chorioallantois interacts intimately with the maternal decidua basalis to form the placenta[2]. In the absence of maternal participation during development, the chick extraembryonic membranes form the sole source of support for the physiological requisites of the embryo including nourishment, gaseous exchange and excretion. For much of the incubation, the CAM, the subject of the current study, is the sole source of gaseous exchange. It is formed of three layers, which include the chorionic epithelium, the mesenchyme and the allantoic epithelium. During growth, blood capillaries and sinuses invade the chorionic layer and become lodged in between the epithelial cells and in so doing they gain close proximity with the ambient air (within 0.2 μm) [3]. In addition the CAM also plays an essential role in osteogenesis by transporting calcium into the embryo from the eggshell [4, 5].

The CAM functions as the equivalent of the placenta in eutherians. It is incipient from embryonic day 4 or 5 (E4 or E5) of incubation [6], and grows into a tripartite membrane consisting of the chorion, the mesoderm and the allantois [7, 8]. Each of the three distinct layers has its definite structural components with specific functions. The chorion is ectodermal in origin and consists of villous cavity cells (VC), capillary covering cells (CC) and endothelial cells (EC) surrounding the lumina of chorionic capillaries [9]. Even in the absence of the shell and shell membranes, the various cell types of the chorion differentiate well, although may be a bit delayed [10]. In the latter study, VC and CC cells, which normally appear by E12-E14, were not apparent until E15. However, later it was shown that presence and transportation of calcium were necessary for proper differentiation of CAM [11].

The CAM mesenchymal layer results from the fusion of the splanchnic mesoderm of the allantois with that one of the chorion and contains blood vessels and stroma[12]. The CAM persists for a duration of 15–16 days until E21 when the chick hatches out and the yolk together with remnants of the CAM become internalized into the abdominal cavity. The CAM completely covers the inner surface of the shell membrane by E12 [13]. During development, vascular capillaries from the superficial aspects of the mesoderm become incorporated into the chorionic epithelium and the specialized cells of the chorion differentiate [9, 12, 14]. A study of the entire incubation period of the CAM showed that by E8, it covers 75% of the inner shell surface rising to 100% by E12 [15]. Towards hatching signs of degeneration have been reported in the cells of the chorionic layer [16].

Study of CAM angiogenesis has indicated that between E5-E7 sprouting is the major mechanism of vessel growth and from E8-E12, intussusceptive angiogenesis preponderates while between E13 to E14, the vasculature expands without increase in complexity [17]. Most CAM morphometric studies use model-based methods [18], which are prone to systematic error (inherently biased) while others are based on two dimensional estimates [19] and none has surveyed the allometric changes of CAM during the incubation period.

It has previously been reported that the rapid phase of angiogenesis is E10 and after initiation of endothelial cytodifferentiation at E14 [20]. Elsewhere, Baum and co-workers found expression of VEGF-A to have peaks at E8-E9 and also at E11-E12 [21] while recently, a peak at E7 and E18 was reported [22].

The CAM is a very useful model in biological and biomedical research for study of angiogenesis [23], tumor growth [24] as well as for cultivation of viruses [25, 26]and helminthes [27]. In angiogenesis and anti-angiogenesis, efficacy and mechanisms of action of pro-angiogenic and anti-angiogenic natural and synthetic molecules has been tried[28]. The CAM itself has been a very useful tool to understand the various modes of angiogenesis during development [29–32]. The usefulness of CAM emanates from its easy accessibility, it is amenable to both intravascular and topical administration of target agents, and can be adapted very easily to study angiogenesis-dependent processes, such as tumor growth. Furthermore, the CAM is easily accessible for both in vivo and ex-ovo studies.

In the current study, development of CAM has been followed from E5 through to E20 using unbiased stereological methods combined with allometric and regression analysis. We have identified three phases of growth, the last one being a phase of regression prior to hatching.

## Material and Methods

### Experimental animals

All techniques employed in this study were approved by the Biosafety, Animal Use and Ethics Committee (BAUEC), of the Faculty of Veterinary Medicine, University of Nairobi. Embryonated eggs from Brown Leghorn layers were procured from commercial breeders and incubated at 37°C and a humidity of 65%. Embryos covering Hamburger and Hamilton stages 19 to 45, which correspond to E8 to E20 of incubation, were obtained and processed according to the methods described below.

### CAM morphometry

At selected time points, 10 embryonated eggs were opened and the embryo together with all the membranes and their contents carefully separated from the shell. Embryos that were 13 days or older were anaesthetized with intraperitoneal injection of sodium pentobarbitone. The CAM was carefully separated from the embryo and yolk sac and washed in phosphate buffered saline (1 X PBS). Subsequently the CAM was immersed in a solution of 2.5% glutaraldehyde in 0.1 M cacodylate buffer (pH 7.4, 350 mOsmol/kgH_2_O). The embryo mass was obtained and after at least 4 hours of fixation, the CAMs were examined under a dissecting microscope and any remnants of shell membranes, albumin or yolk carefully cleaned off with fine forceps. Total volume of CAM was obtained by Scherle method of weight displacement [33]. Morphometric analysis was performed on 4–5 CAMs from the best preserved specimens.

### CAM sampling

Fixed CAMs were placed on wax plates and completely diced into quadrats measuring roughly 2 x 2 cm. The first quadrat was picked at random (either 1 or 2) and then every other second quadrat was selected. The slice was then divided into two halves, one of which was processed for light microscopy (LM, paraffin embedding) while the other one was processed for transmission electron microscopy (TEM).

### Microscopy

For LM and TEM CAMs were cut into smaller slices and post-fixed in osmium tetroxide; block-stained using uranyl acetate, dehydrated through ascending concentrations of ethanol, and embedded in epoxy resin. Semithin sections were obtained at a nominal thickness of 1 μm, stained with toluidine blue, and viewed under a Leica DMBR digital light microscope. Ultrathin sections were obtained at 90 nm, counterstained with lead citrate, and viewed on a Philips CM-12 transmission electron microscope or Phillips TEM 400.

### Immunohistochemistry

Specimens for immunohistochemistry for evaluation of proliferation (PCNA) and apoptosis (TUNEL) were fixed in 2% paraformaldehyde for 1 h, rinsed in 15% sucrose and then stored in 70% ethanol. The specimens were dehydrated though a graded series of ethanol, embedded in paraffin wax sections obtained at a thickness of 5μm.

### PCNA staining

After deparaffinization and rehydration of the paraffin sections, antigen retrieval was performed by microwaving the sections in 10 mM sodium citrate buffer (pH 6.0) at maximum power for 2 min and at 180W for 13 min. The slides were cooled at ambient temperature for 30 min, washed three times for 5 min in TBS and incubated with 1% bovine serum albumin (BSA) blocking solution in TBS for 10 min at in a humidified chamber. To detect PCNA, sections were incubated with a monoclonal mouse anti-human PCNA antibody (ab29, Abcam, Cambridge, UK) diluted 1:500 in DAKO antibody diluent and treated with background reducing buffer (S3022, DAKO, Baar, Switzerland) overnight at 4°C in a humidified chamber. The following day, the sections were washed three times in TBS for 5 min each and incubated with biotinylated anti-mouse secondary antibody (1:500) in 1xTBS, (BA-9200, Vector Labs Inc., CA, USA) for 45 min. After three washes in 1xTBS for 10 min each, the sections were incubated with avidin biotinylated horseradish peroxidise complex (PK-4000, Vectastain ABC-Peroxidase Kits, Vector Labs Inc., CA, USA) for 45 min followed by three washes in 1xPBS, each for 10 min. The sections were incubated with DAB (3,3’-Diaminobenzidine, (K3468, DAKO, Baar, Switzerland) chromogen-substrate buffer solution according to the manufacturer’s instructions (1 drop of DAB chromogen per 1 ml of substrate buffer) for 5–10 min until a brown signal was detected. The sections were immersed in 1xPBS to stop the reaction and then washed for 2 min under running water. Counterstaining was performed using Mayer’s Hematoxylin for 2–5 min and the sections were washed for 10 min under running tap water.

### TUNEL staining

Dewaxed sections were rehydrated, and antigen retrieval achieved by incubating the sections with 15 μg/ml Proteinase K in 10 mM Tris-HCL at pH 7.5 (03115887001, Roche, Basel, Switzerland) for 30 min in a humidified chamber. The sections were washed three times in PBS for 10 min each, incubated with freshly prepared TUNEL reaction mixture (TUNEL Enzyme solution diluted 1:12.25 in TUNEL Label solution, (11684817910, Roche, Basel, Switzerland)) for 60 min at 37°C in a humidified incubator. Subsequently sections were incubated with anti-fluorescein antibody conjugated to horseradish peroxidise, TUNEL-POD (1772465, Roche, Basel, Switzerland) diluted 1:2 in PBS, for 30 min at 37°C in a humidified incubator. The sections were washed three times in PBS for 10 min each, incubated with DAB chromogen-substrate buffer solution (K3468, DAKO, Baar, Switzerland) for 5–10 min until a brown signal was detected and immersed in PBS to stop the reaction. The sections were washed for 2 min under running distilled water and counterstaining as above

### Morphometric estimations

Estimation of the morphometric parameters of the various changing components of the CAM was done using Cell^D imaging software (Olympus Europa GmbH, Hamburg, Germany). These parameters included volume densities of CAM components using point counting methods (chorion, mesenchyme and allantois) as well as the components of the chorion (chorionic capillaries and chorionic cells).

#### Volume densities and volumes

The volume density of a component of interest Vvc, for example was calculated from the equations:

$$\;Vvc=Pc\;*\;Pt^{-1}$$

Where Pc and Pt are point counts on profiles of the component of interest and total number of points on profiles of the reference space, respectively. Then Vc, the volume of the component of interest is estimated from the formula;

Vc=Vvc * V(ref)

where V(ref) is the volume of the reference space.

Differential count of points on the targeted constituent components of CAM were also done to obtain their respective volume densities and subsequently, their absolute volumes.

#### Data analysis

Statistical analysis was performed to pick differences among the volumes of CAM as well as the proportions of the various CAM components and their absolute volumes at selected time points. To achieve this, Microsoft Excel 2010 was used to perform single factor ANOVA to detect significant differences. Scheffe’s test was used to perform posthoc analysis of the targeted values using Graphad Software free online at: (http://graphpad.com/quickcalcs/posttest2/).

To determine trends in the various changing parameters, least squares regression analysis was performed and the coefficient of variation (r) estimated to determine the goodness of fit. All non-ratio data were log-transformed and the power equation,

$${\text{log}}_{10}Y={\text{log}}_{10}a\;+\;b{\text{log}}_{10}X$$

was employed to calculate the scaling exponent (b) and hence the relevant allometric equation was obtained.

### Molecular data analysis

Detection of expression levels for VEGFA, HIF1α, VEGFR2 and SDF-1 during development of CAM was done on selected time points. Total RNA was extracted from CAM samples, collected and frozen before analysis, at the following time points: E8, E11, E13, E15, E18 and E20. RNA was purified using High Pure RNA Tissue Kit (Roche Diagnostics GmbH, Manheim, Germany). The RNA concentrations were determined spectrophotometrically. cDNA was synthesized by reverse transcription (1 hour at 37°C) using 500 ng of total RNA, 2.5 μM of the oligo-dT primers and 10U of Reverse Transcriptase (Transcription High Fidelity cDNA Synthesis Kit, Roche Diagnostics GmbH, Manheim, Germany). The cDNAs were further examined using RT^2^ qPCR Primer Assays for Chicken (Chicken QuantiTest Primer Assay) with the following commercially available primers: QT01140244 for KDR2 (VEGFR2), QT00593327 for VEGFA, QT00596309 for HIF1α and QT01139026 for SDF-1 (CXCL12). Quantitative PCR was performed using Real Time PCR (Applied BioSystems 7500 Fast). The total volume of each reaction was 50 μl with less than 100 ng cDNA per reaction. The amplification conditions of each RT-PCR cycle were as follows: denaturation reaction at 94°C for 15 sec, primer annealing step at 55°C for 30 sec, followed by primer extension– 72°C for 30 sec. The expression levels of the selected genes were normalized to GAPDH (QT00588973) using ΔCT calculations. In order to avoid contamination, we have included an internal non-template control in each run. The fold change in expression levels of cDNA were determined from the ΔCt values obtained as compared to ΔCt values of time point E8 (see S1 Fig).

### Critique of the methods

Estimations of the absolute volumes of CAM cannot be procured with absolute certainty since some tiny pieces of the shell membrane or even minute aggregations of albumin or yolk may be missed under the light microscope. Furthermore, tiny breakages in the epithelium of the chorion were not uncommon. These problems were overcome by selecting the best preserved specimens from a group of ten. At E8, not all the CAM layers are fused, which means that the absolute volume was a slight overestimate since it also included the contributions from allantoic and chorionic layers that were not fused.

## Results

**Fig 1:**
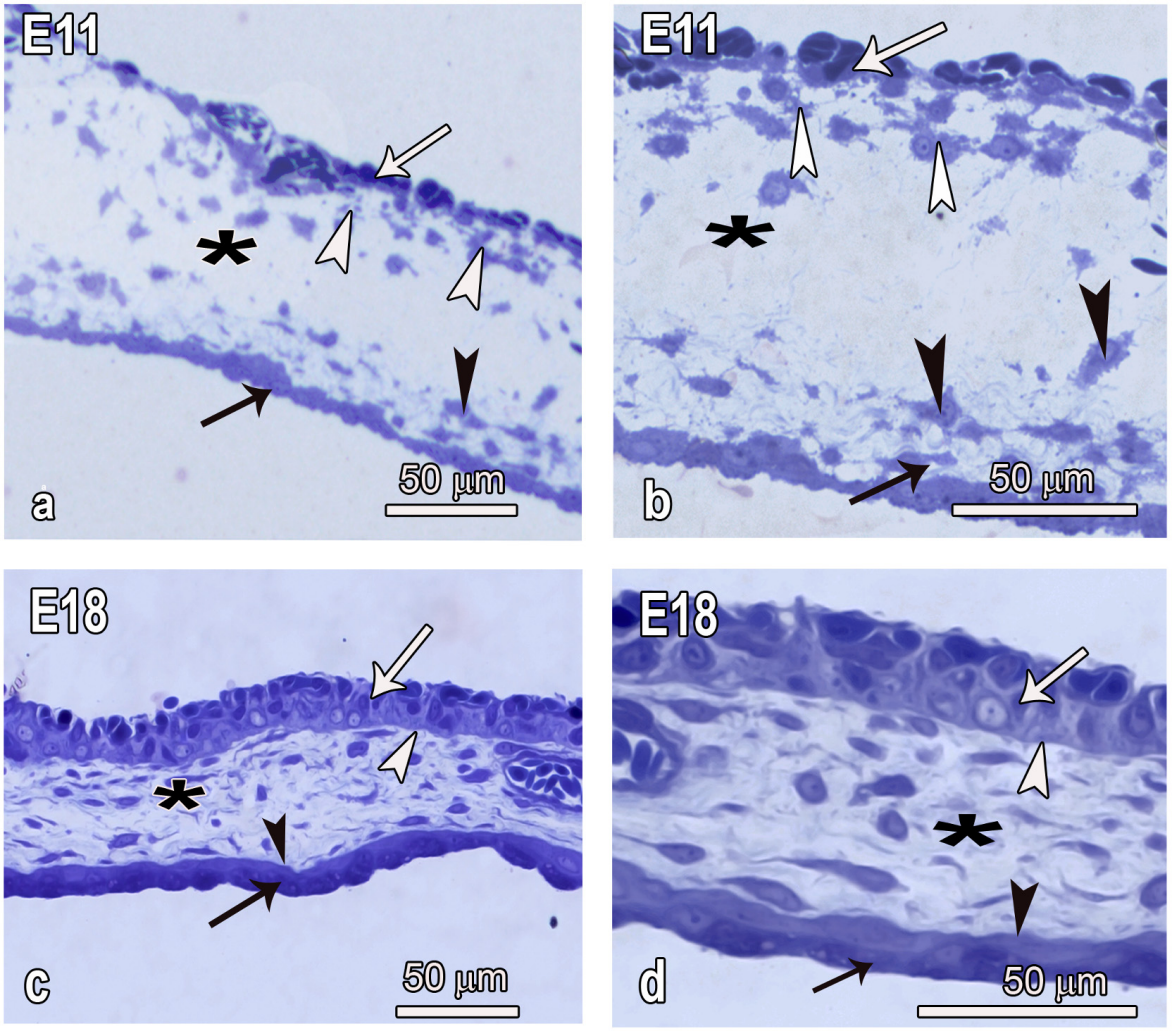
Semithin micrographs showing the distribution of cells within the various layers of the developing CAM at E11 and E18. **a and b:** Both the chorion (white arrows) and the allantois (black arrows) are relatively thin early during development and there are numerous cells concentrated in the subepithelial layer of the chorion (white arrowheads) and also in the subepthelial layer of the allantois (black arrowheads). Note both the chorion and the allantois are of irregular thickness. In the middle part of the mesenchymal layer (asterisks), cells are few. **c and d:** At E18 the CAM has reached maturity and the chorion (white arrows) and the allantois (black arrows) are both 3–4 times thicker, have an almost uniform thickness and their basal aspects are delineated by a prominent basal membrane (white and black arrowheads respectively). Distribution of cells in the mesenchymal layer (asterisk) is almost uniform.

**Fig 2:**
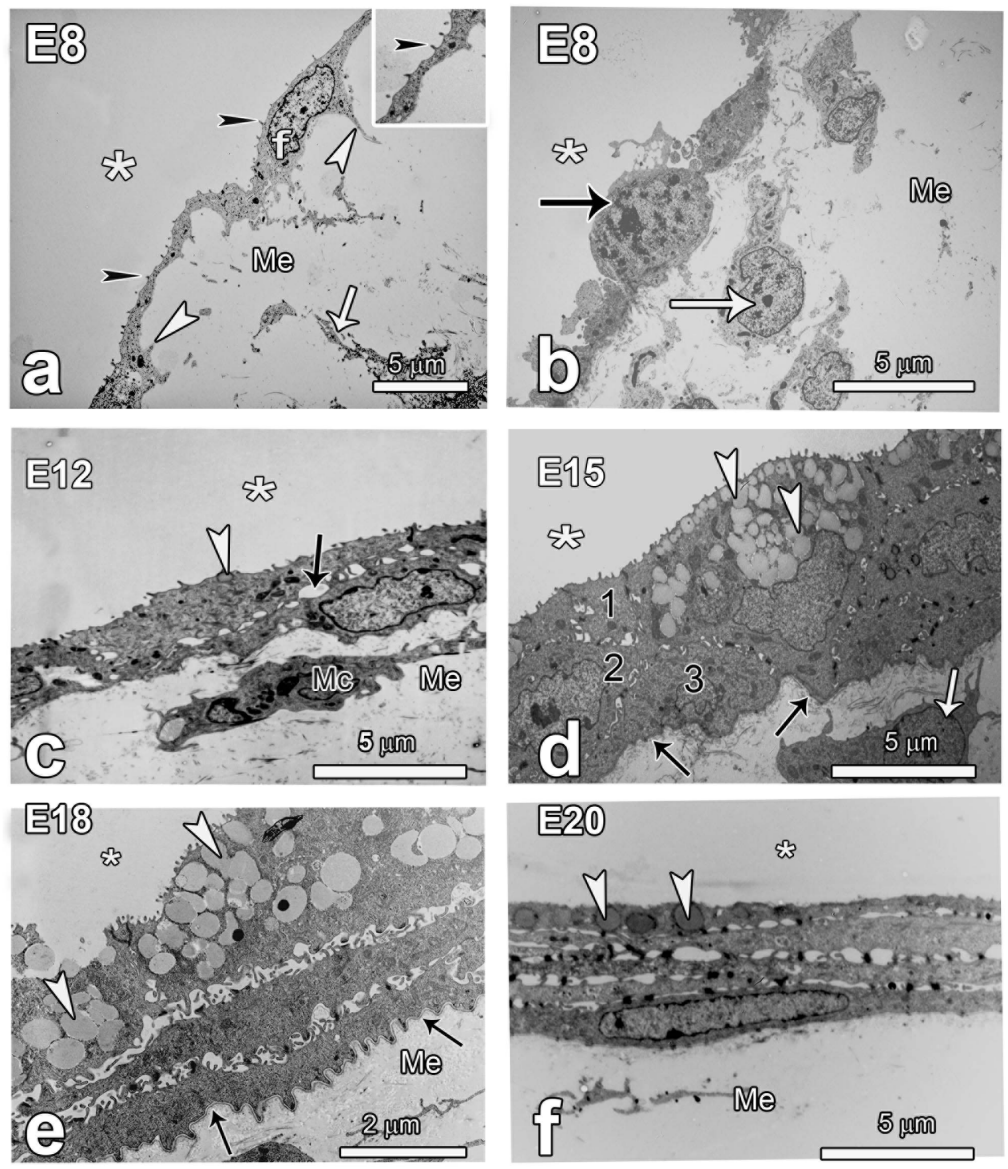
TEM micrographs showing the changing ultrastructure of the allantoic layer between E8 and E20. Note that in all cases the microvilli project into the allantoic lumen (asterisks) and this is important in identifying the mesenchyme (Me) in cases where mesenchymal cells are not evident. **a and b**: The allantois at E8 consists of a single layer of squamous fibroblast like cells (f) with numerous filopodia (white arrowheads) extending into the mesenchyme and short microvilli (black arrowheads) towards the allantoic lumen. The mesodermal layer has loose connective tissue and fibroblasts (white arrows). Some cells in the allantois show mitotic figures (black arrow in B). **c and d:** Thickening of the allantois is notable by E12 where it starts to recruit cells to form a two cell layer separated by enormous intercellular spaces (arrow in c). The first few granules start to appear in the outer layer of the allantois (arrowhead in c). A mesenchymal cell (Mc) is closely aligned with the inner layer of the epithelium. By E15, a two-cell layer is well accomplished and formation of the third layer begins (see layers 1, 2, 3 in d). Notice the granules in the outer layer of the allantois (arrowheads) are well developed. The basal border looks irregular and the basement membrane is amorphous (black arrows) and there are cells close to the epithelium (white arrow). **e and f:** The allantois has 3–4 well-formed cell layers at E18 and the basement membrane (black arrows) is neatly formed. The outer cell layer has abundant glycogen granules (arrowheads) and short microvilli. By E20, the layers of the allantois look shrunken and the glycogen granules (arrowheads in f) are depleted.

**Fig 3:**
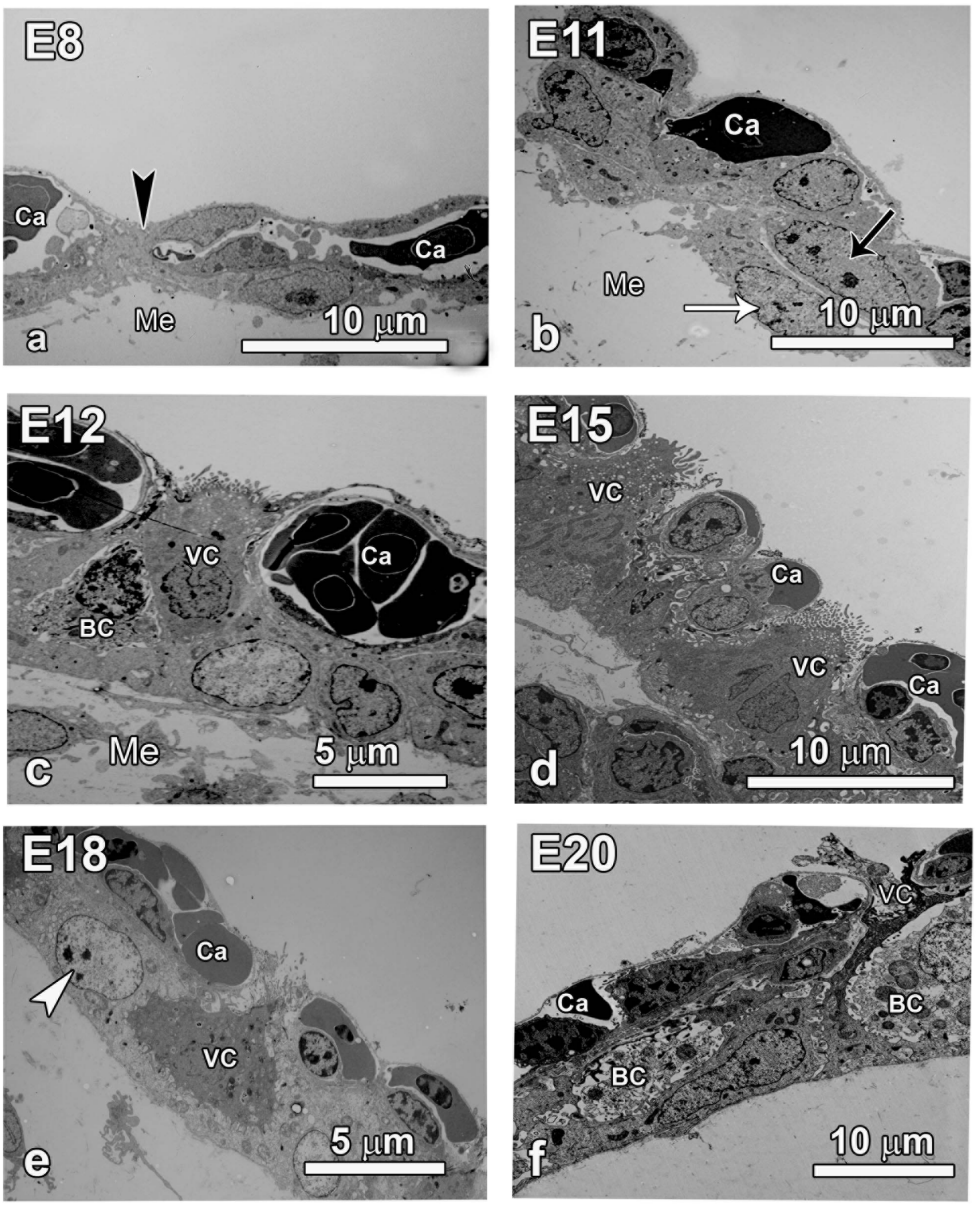
TEM micrographs showing the changing structure of the chorionic layers. **a and b**: The chorion at E8 is thin with large capillaries (Ca) separated by undifferentiated epithelial cells (arrowhead in a). The mesenchyme (Me) is also shown. By E11 there is recruitment of cells from the subchorionic layer of the mesenchyme (Me). Such cells (white arrow) are loosely attached to primary cells of the chorion (black arrow in b). At these stages the various cell types of the chorionic layer are not differentiated. **c and d**: The typical chorionic cells such as the villous cavity (VC) cells and basal (BC) cells become recognizable by E12 and by E15 (d) such cells are well differentiated. The chorionic capillaries (Ca) remain on the external aspect of the epithelium. **e and f**: By E18 the first signs of degeneration are evident in the VC, with loss of microvilli although some cells in the basal layer show some mitotic activity (arrowhead). Clear signs of cell degeneration at E20 include dissolution of basal cells (BC) and crumbling of VC cells.

**Fig 4:**
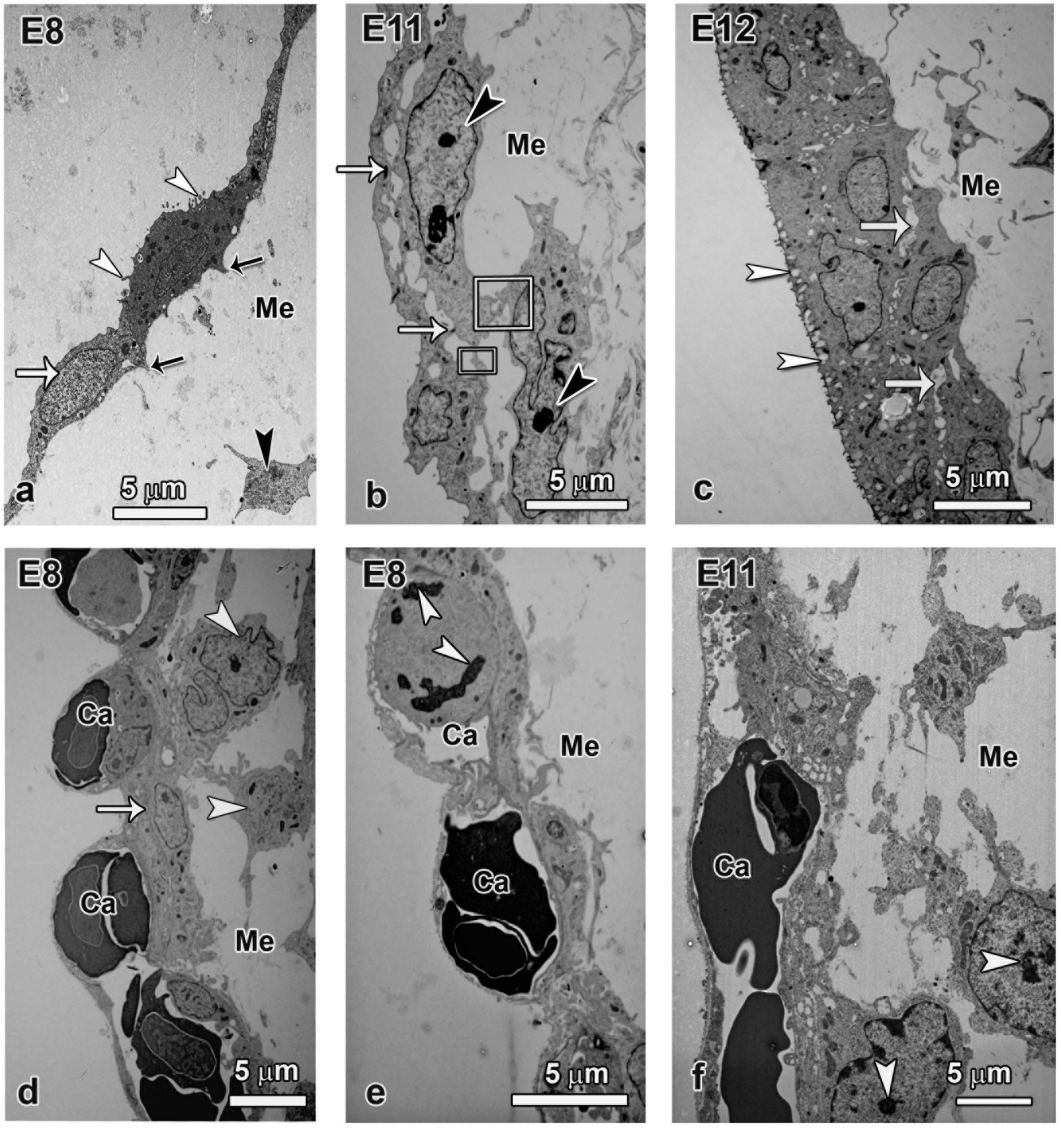
TEM micrographs showing cell layer augmentation in the allantois (a-c) and the chorion (e-f). The mesenchyme (Me) and capillaries (ca) are denoted wherever applicable. **a–c:** The allantois is made up of a single layer of cells (white arrow) with scanty short microvilli (white arrowheads) at E8 and the mesenchyme has few cells (arrowhead) and cell aggregations in the subepithelial layer of the mesenchyme are not apparent. Notice the filopodia projecting into the mesenchyme (black arrows). By E11 the allantoic subepithelial layer is invaded by cells (arrowheads) with processes (rectangles) that attach on the epithelial layer and now intercellular spaces are established as well as close cell contacts (arrows in b and c). The allantois is made up of two cell layers by E12 and the outer layer begins to accumulate granules (arrowheads in c). **d–f:** In the chorion at E8, a single layer of cells (arrow) is evident but it is associated amorphous subepithelial cells (arrowheads) that appear to attach to the epithelium. Furthermore cell division is evident even in the vascular endothelium (arrowheads in e) showing robust growth of the capillaries (ca). By E11 some cells from the mesenchyme have closely adhered to the epithelium and many of such cells in the subepithelial region have mitotic figures (arrowheads in f).

**Fig 5:**
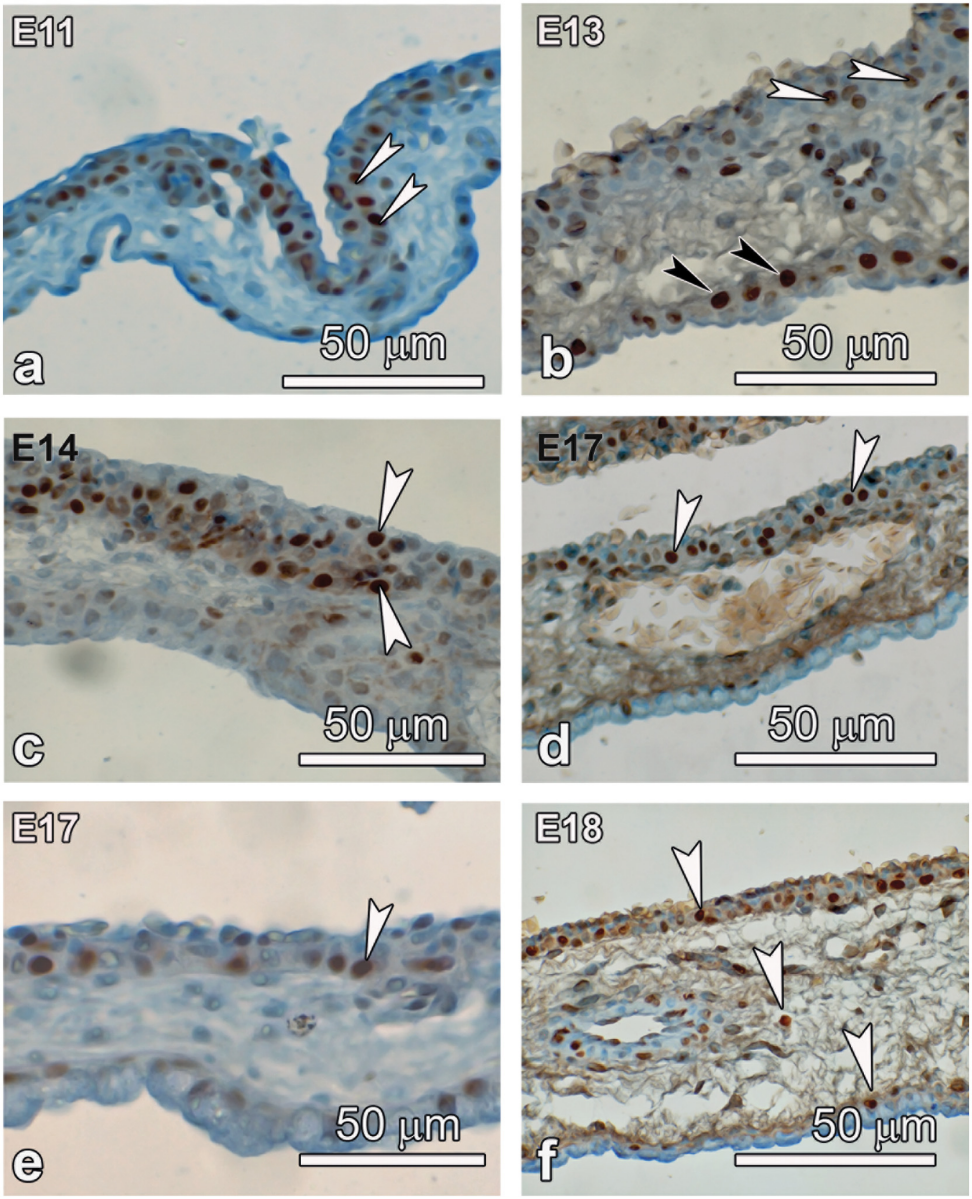
Light micrographs showing cell proliferation (a, c, e) and apoptosis (b, d, f) in the developing CAM. a, c, e: Cell proliferation in the chorionic epithelium at E11 but mainly in the subepithelial layer. By E14 the proliferation is now preponderant within the chorion and subchorionic rare and by E17; only occasional proliferating cells are encountered in the basal layer. b, d, f: Cell apoptosis is more pronounced in the allantois from E13 but mild in the chorion, and shifts to chorion by E17 and is quite pronounced by E18.

**Fig 6:**
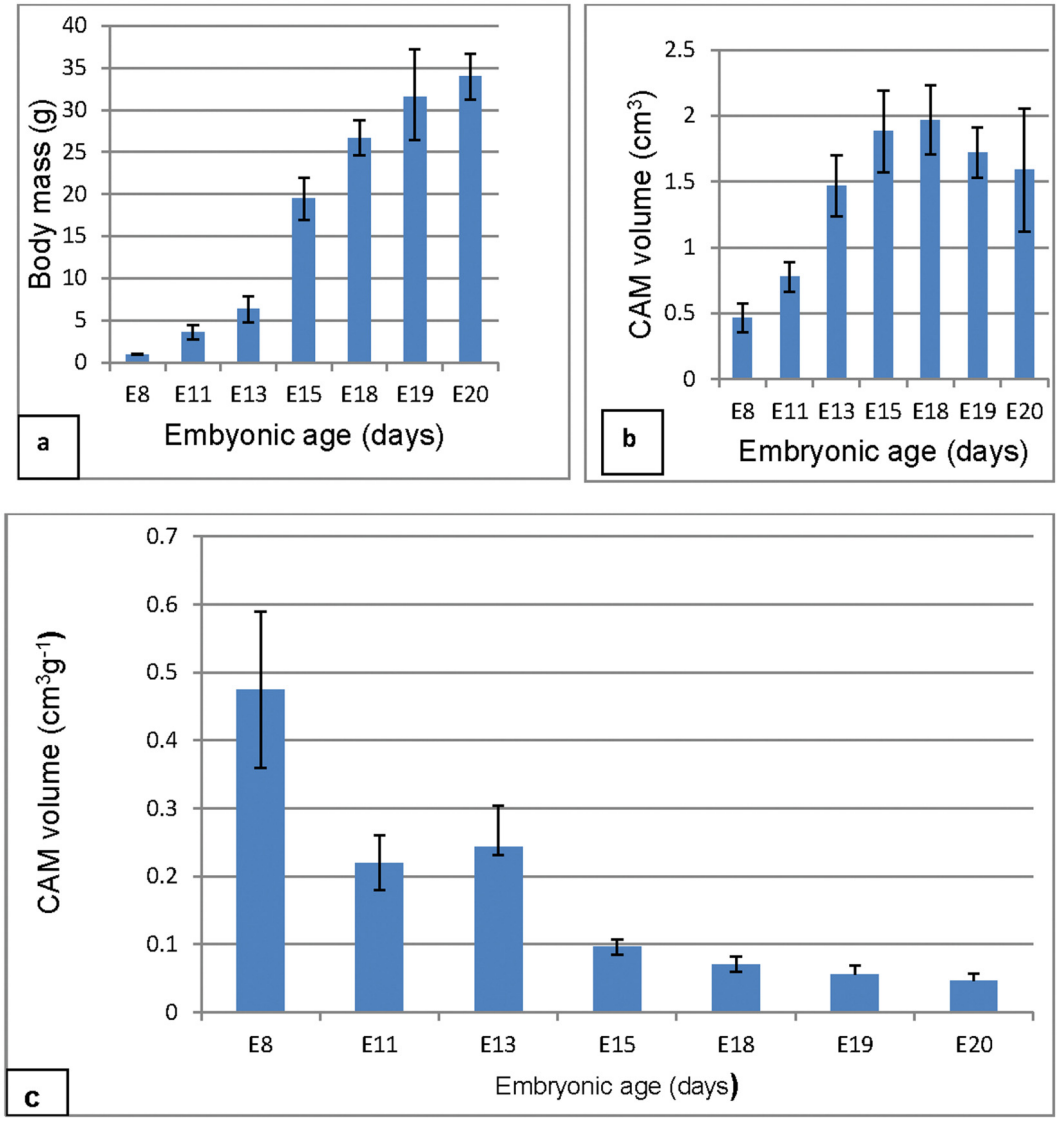
Bar graphs showing the variation in body mass (g), CAM volume (cm^3^) and body mass standardized CAM volume (cm^3^g^-1^). **a**: Notice that body mass increases steadily between E8-E13, sharply between E13-E15 and then steadily between E15-E20, marking the three growth phases. **b**: The CAM volume increases fast between E8-E13, steadily between E13-E15 and then declined between E18-E20, the latter was thus a phase of regression. The phase of rapid CAM growth precedes that of embryonic growth. On Scheffé's test (p ≤0.05) CAM volume at E8 and E11 were significantly lower than at all the other subsequent days (asterisks). **c:** Body-mass standardized CAM volume decreased gradually through the entire period.

**Fig 7:**
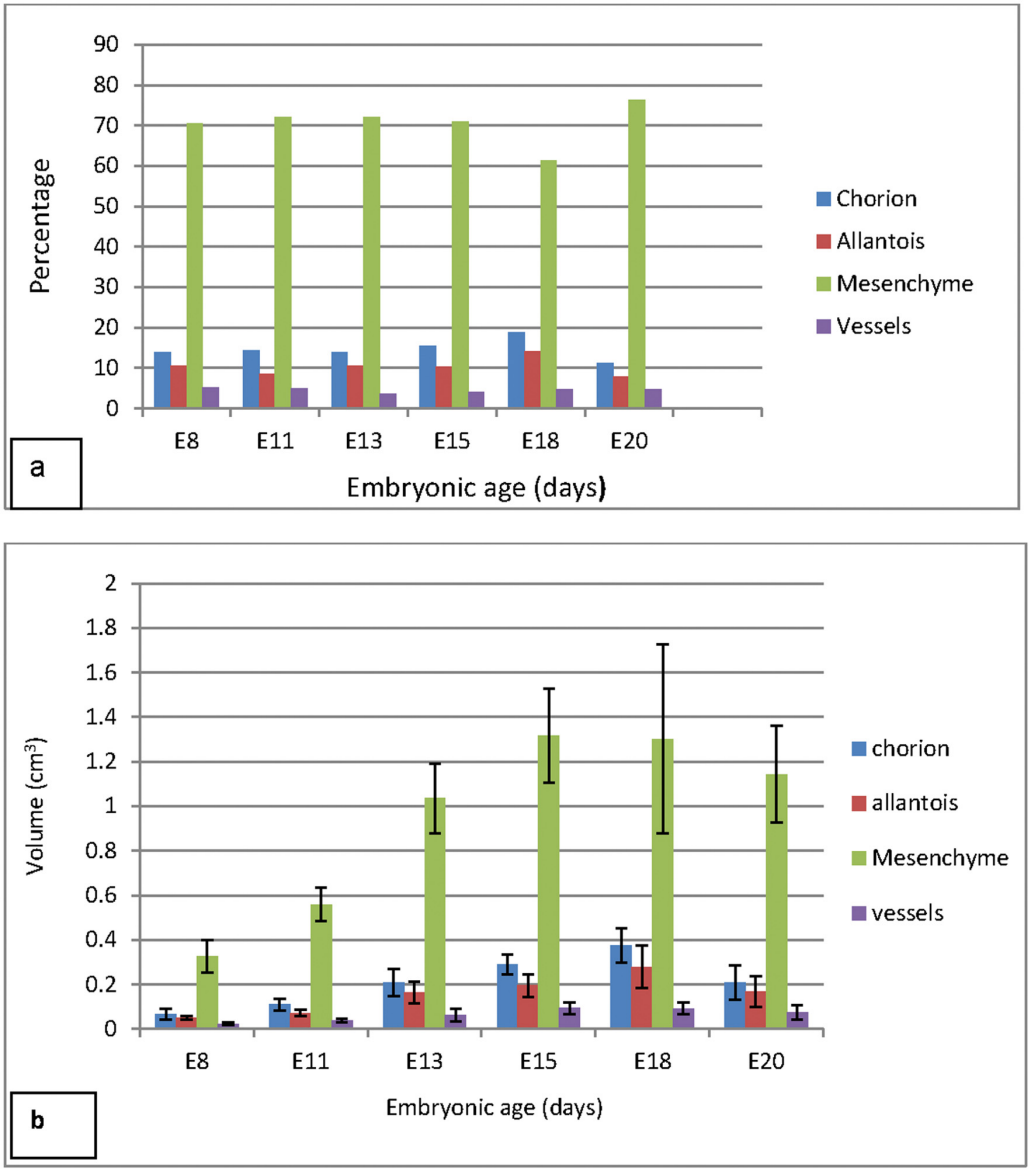
Bar graphs showing the proportions (a) and absolute volumes (b) of the CAM components. **a:** The mesoderm remained the largest component during the entire growth period at about 70%, declining slightly at E18. The chorion was between 10–18%, being highest at E18 and decreasing to about 10% at E20. The allantois remained at around 10%, peaked at E18 to above this value and then declined to about 8% by E20. The proportion of the large vessels appeared to remain steady at about 4–5% of the CAM. b: In absolute terms, the values of the components followed closely the trend of the proportions. The highest value for the mesoderm was at E15 while the chorion and allantois peaked at E18. Volume of chorion at E8 was significantly lower than all the other time points except E11. The volume at E11 was lower than at E15 and E18. Similarly, chorionic volume was significantly lower at E13 and E20 than at E18. Allantoic volume at E15 and E18 was significantly higher than at both E8 an E11while mesenchymal volume remained significantly higher in the ages E13-E20 than at E8 and E11. Large vessels at E15 and E18 had significantly greater volumes than at E8 and E11.

**Fig 8:**
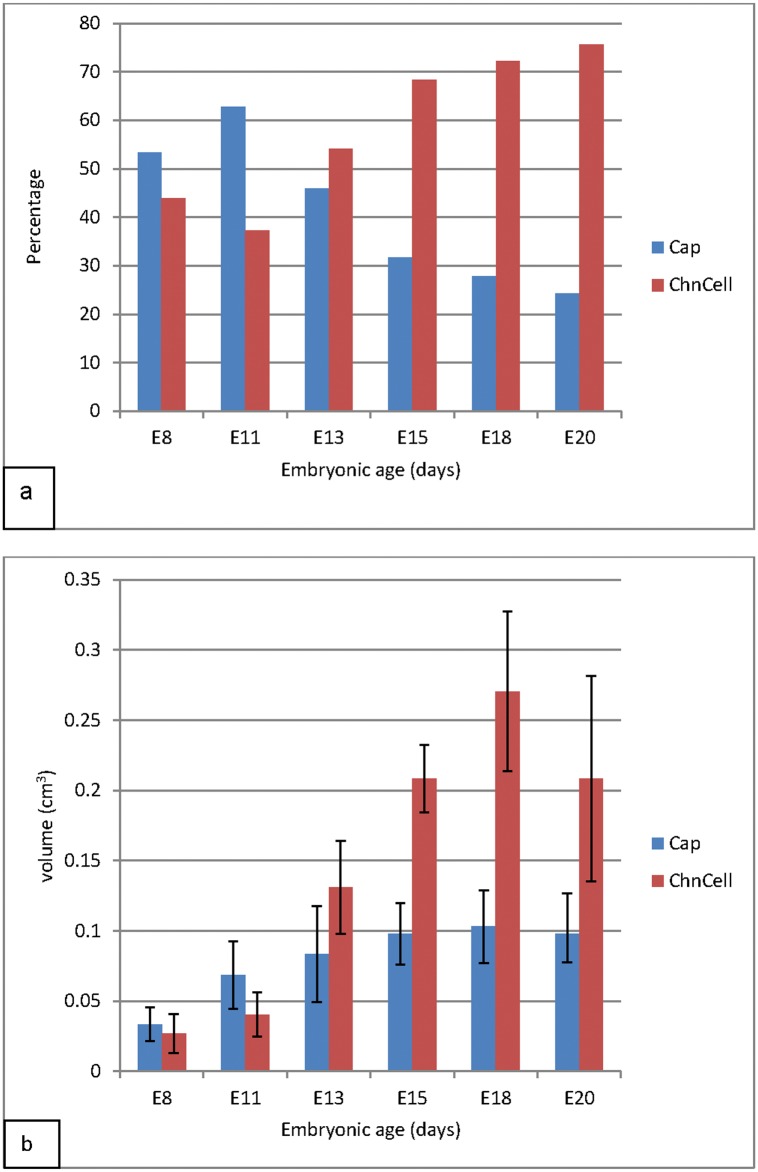
Bar graphs showing the proportions of the capillary and non-capillary components of the chorion (a) as well as the respective absolute volumes (b). **a**: Between E8 and E11, the chorionic capillaries take over 50% of the chorionic volume, but are overtaken by chorionic cells as from E13. By the end of incubation, the capillaries take only about 25% of the chorionic volume. **b**: In absolute terms, the values of both the capillary and non-capillary components reach a peak at E18 and then begin to decline. The volume of the capillaries remains the greater component up to E11. Volumes of the chorionic capillaries were significantly greater (P≤ 0.05) at E15 and E18 than at E8. Chorionic cell volumes were significantly greater (P≤ 0.05) at all subsequent time points than at E8 and were greater also than those at E11 except at E13. Volume was significantly greater at E18 than at E13.

**Fig 9:**
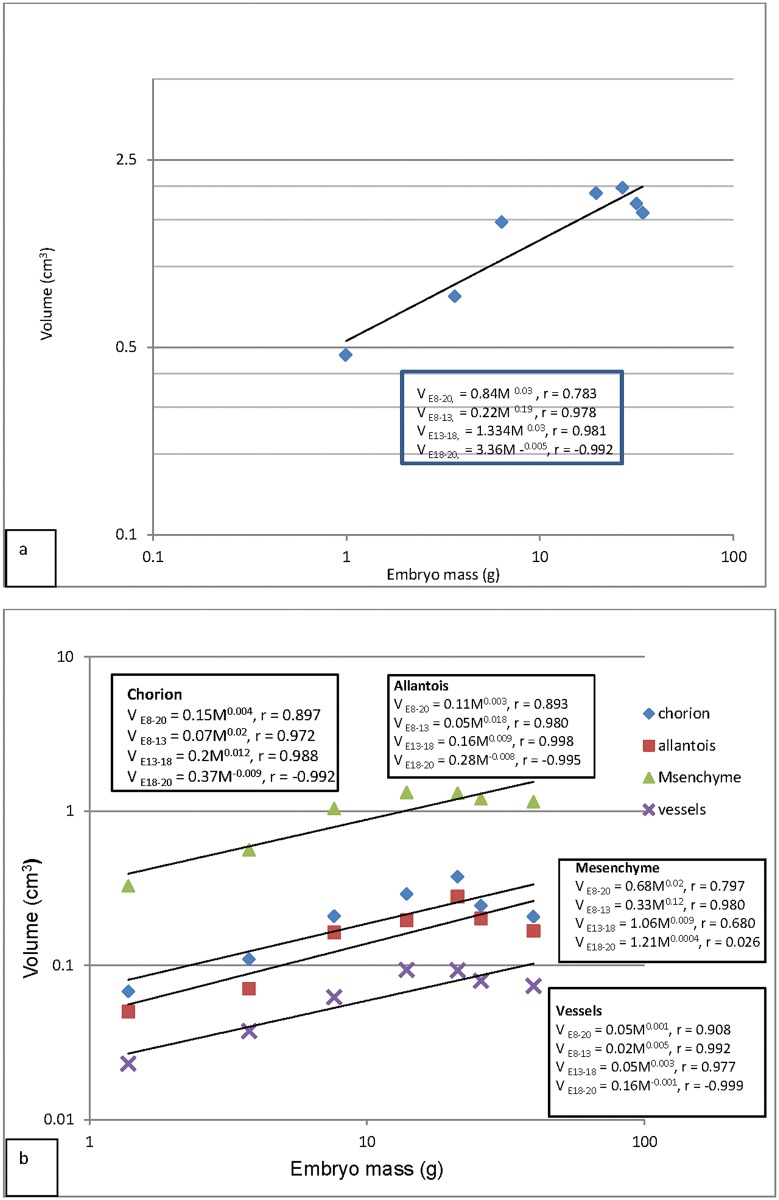
Logarithmic line graphs showing growth rates of CAM (a) and the various coarse components of the CAM (B). **a:** The CAM grew rapidly in phase I (E8-E13), moderately at phase II (E13-18) and was undergoing regression in phase III (E18-E20). CAM volume was significantly correlated to body mass (P = 0.01). CAM growth was strongly positively related to body mass increase up to E18, but it had a strong negative correlation until time of hatching. **b:** Logarithmic line graphs showing growth regression analysis of the coarse CAM components. The mesoderm was the fastest growing component while the large vessels were the slowest. Both the chorion and allantois grew at the same rate. When viewed on individual basis at various growth phases, all components were growing fastest in phase I and except the mesoderm, they were regressing in phase III. All components were significantly correlated to body mass during the entire period (P ≤ 0.05). The chorion, allantois and vessels were regressing in phase III (showed a strong negative correlation with body mass).

**Fig 10:**
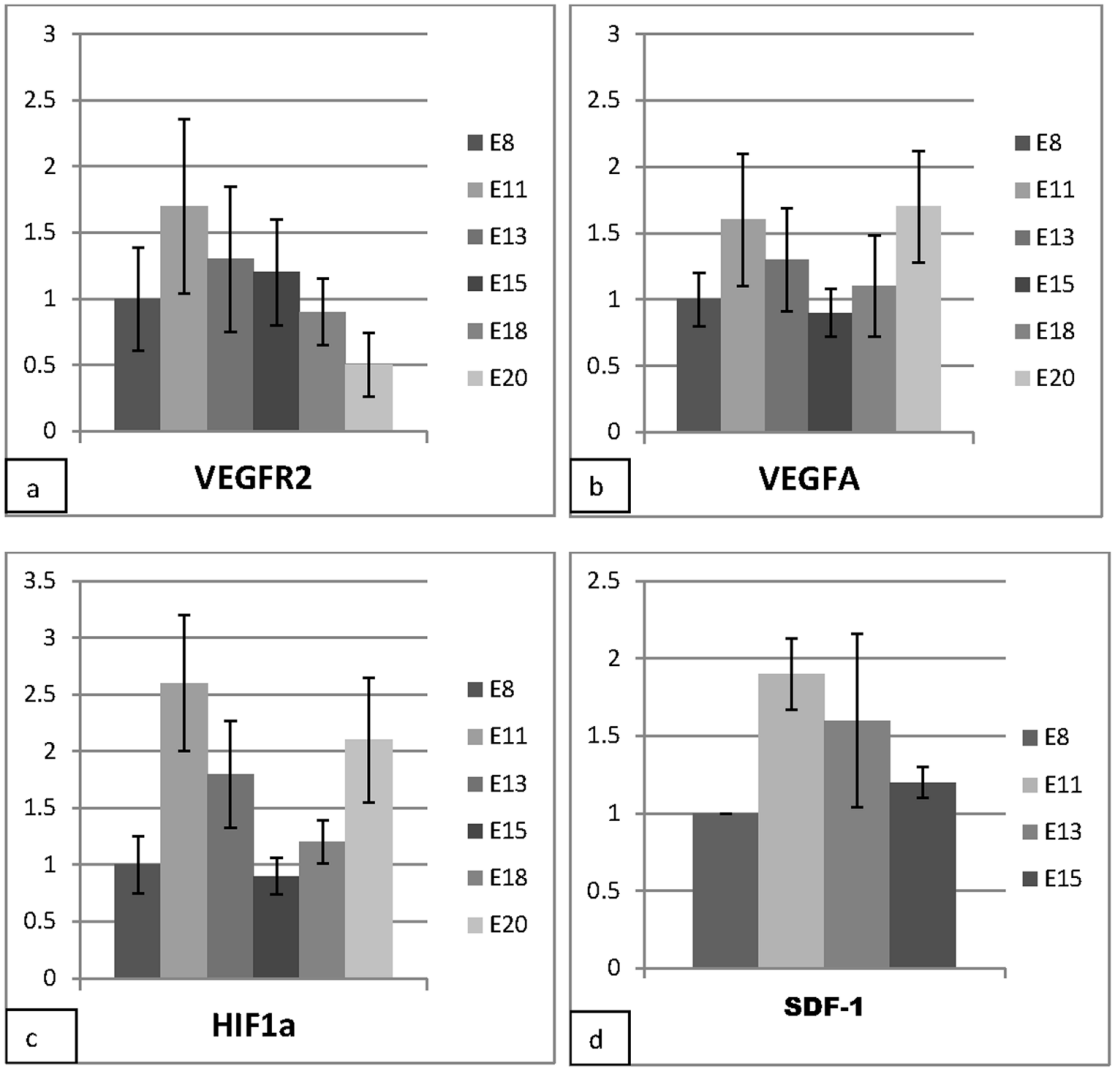
Relative expression levels of selected angiogenesis related molecules during the course of CAM development. The expression levels, however, did not show any statistically significant differences among various time points (single factor ANOVA). **a**: Vascular endothelial growth factor receptor 2 (VEGFR2), the vessel endothelial marker, reaches a peak at E11, and then decreases steadily, being least at E20. **b:** There is an increase in the expression levels of HIF1α at E11, a sharp decrease towards E15 with a peak at E20. **c:** VEGFA peaks at E11 and again at E20. Its lowest expression levels are at E15 **d:** The stromal derived factor 1 (SDF-1) expression levels reach a peak at E11 and decline towards E15.

The coarse components of the CAM investigated in the current study include the chorion, the mesenchyme, the allantois and the large (conducting) blood vessels. The latter vessels were identified as those that were ≥25 μm in diameter. The growth of the CAM and its components was followed from E8, through to E20 using light microscopy (Fig 1), ultrastructural analysis (Figs 2–5) as well as immunohistochemistry (Fig 6). Development of the CAM was divided into three phases based on allometric and regression analysis (see Figs 7–9). Relative expression revels of selected angiogenesis related molecules were accomplished with RT-PCR (Fig 10).

Early during development, both the chorion and allantois were thin layers but in both cases the subepithelial portions of the mesoderm had aggregations of cells (Fig 1). In contrast, the middle layer of the mesenchyme had only a few cells. A basement membrane was not apparent and the basal outline of the epithelial layers in both cases was irregular. Both layers had transformed tremendously by E18 in that they were much thicker, a prominent, well-outlined basement membrane was evident and the basal outline appeared smooth. Distribution of cells in the mesenchyme was uniform and subepithelial cell aggregations were no longer evident (Fig 1).

Development and augmentation of the allantois was further closely followed at TEM. At E8 the allantois consisted of a single layer of squamous fibroblast-like cells with filopodia extending into the mesenchyme and short scanty microvilli projecting towards the allantoic lumen (Fig 2). Some cells in the allantois showed mitotic figures, as did those close to the single-layered epithelium (Fig 2). By E12 the allantois started to recruit cells to form a two cell layer separated by enormous intercellular spaces and the first glycogen granules were evident in the outer layer. Formation of the third layer was evident at E15. The outermost layer of the allantois at this stage had more glycogen granules and short microvilli and by E18 the allantois had 3–4 well-formed cell layers and the basement membrane was remarkably conspicuous with numerous infoldings. The outer cell layer had abundant, occasionally coalescing granules and short microvilli. Signs of degeneration were evident at E20, with notable shrinkage of the layers, depletion of the glycogen granules and loss of microvilli.

The chorion developed following steps similar to those of the allantois, albeit without formation of distinct cell layers. It was thin with large capillaries at E8, but the various cell types characteristic of the chorion were not differentiated (Fig 3). There was recruitment of cells from the ‘subchorionic layer’ of the mesenchyme by E11 and such were seen to be loosely attached to primary cells of the chorion thus leaving large intercellular gaps. At this stage the thickness of the chorionic layer was relatively increased compared to the situation at E8. Differentiation of the phenotypically unique cells of the chorion such as the villous cavity (VC) cells, capillary covering (CC) cells and basal (BC) cells was evident as from E12 and by E15 such cells were well differentiated and mature (Fig 3). The first signs of degeneration of the chorion were evident by E18 as loss of microvilli in the VC cells although some cells in the basal layer showed some mitotic activity, indicating that some cells retained proliferative activity even in the last phase of development. Dissolution of basal cells and crumbling of VC cells was preponderant at E20 (Fig 3). Notably, the chorionic capillaries formed the middle core of the chorion at E8 but as the chorion grew from the mesenchymal side, the capillaries remained at the external part of the chorion.

To examine the events leading to the increase in the thickness of the chorion and allantois, the two layers were closely examined at ultrastructural level (Fig 4) and cell dynamics investigated by cell proliferation and apoptosis assays (Fig 5). The allantois at E8 was made up of a single layer of cells joined end to end. Such cells were devoid of glycogen granules but hard scanty, short microvilli projecting into the allantoic lumen. The mesenchymal cells were seen to be loosely attached to primary cells of the allantois, thus leaving large intercellular gaps or the attachment was effected through elongated cell processes (Fig 4).

Association of some mesenchymal cells with the simple single layer of the chorion was evident as early as E8, either directly or through filopodia (Fig 4). At this time, mitotic figures were evident in the capillary endothelial cells showing that capillary growth was concomitant with augmentation of the chorion. Recruitment of mesenchymal cells was accelerated by E11 and cells with mitotic figures were observed either in contact with the chorine or in close proximity.

Cell dynamics were investigated by studying cell proliferation and apoptosis in the CAM (Fig 5). Early during development, cell proliferation was noted to be robust in the subepithelial layer of the chorion, where cells appeared to be recruited from the mesenchymal layer. This was most evident at E11. By E14 much of the proliferation was within the chorion and by E17, only occasional proliferating cells were encountered in the basal layer. In contrast, mild, cell apoptosis was noted first in the allantois at E11 and was quite pronounced by E13 (Fig 5). Apoptosis was seen to be remarkably increased in both the chorion and allantois at E18 and some apoptotic cells were also encountered in the mesenchyme.

### Morphometry

#### Body mass and CAM volume

Variations in the embryonic body mass, CAM volume and body mass-standardized CAM volumes were monitored throughout the incubation period (Fig 6). Body mass increased sharply between E8-E13, steadily between E13-E18, and then slowly between E18-E20. In phase I (E8-E13), embryo mass increased by 540%, in phase II (E13-E18) the value was 321% while in phase III (E18-E20) it was 27% (Table 1). Absolute CAM volume (Table 2) increased from a low of 0.47 ± 0.11 cm^3^ at E8 to a high of 2.05 ± 0.27 cm^3^ at E18, and then decreased to 1.6 ± 0.47 cm^3^ at E20.CAM volumes and volumes of the coarse CAM components are presented in Table 2. In phase I (E8-E13) CAM volume increased by 89%, in phase II (E13-E18) the values was 34% while in phase III (E18-E20) it decreased by 38% (Table 1).On Scheffé's test (p ≤0.05) CAM volume at E8 and E11 were significantly lower than at all the other subsequent days.

**Table 1:** Growth phases and percentage changes in body mass and CAM volume in the developing chick embryo.

Phase	Age ranges	Body mass range (g)	Increase (%)	CAM volume range (cm^3^)	Increase (%)
Phase I	E8-E1	0.99–6.34	540	0.776–1.468	89
Phase II	E13-E18	6.34–26.69	321	1.468–1.968	34
Phase III	E18-E20	26.69–33.96	27.2	1.968–1.588	-38

**Table 2:** Body mass, CAM volumes and volumes of the various coarse CAM components in the developing chick embryo. Values are means ± SD.

		Volumes (cm^3^)				
Age (days)	Mass (g)	CAM	Chorion	Mesenchyme	Allantois	vessels
E8	0.993 ± 0.063	0.468 ± 0.109	0.07 ± 0.024	0.33 ± 0.07	0.05 ± 0.01	0.02 ± 0.001
E11	3.63 ± 0.84	0.776 ± 0.111	0.11 ± 0.027	0.56 ± 0.08	0.07 ± 0.01	0.04 ± 0.001
E13	6.34 ± 1.56	1.468 ± 0.233	0.21 ± 0.06	1.03 ± 0.16	0.16 ± 0.05	0.06 ± 0.029
E15	19.47 ± 2.53	1.882 ± 0.31	0.29 ± 0.06	1.31 ± 0.21	0.2 ±0.05	0.09 ± 0.027
E18	26.67 ± 2.09	1.968 ± 0.265	0.37 ± 0.08	1.3 ± 0.42	0.28 ± 0.1	0.09 ± 0.027
E19	31.52 ± 5.69	1.722 ± 0.19	0.24 ± 0.08	1.2 ± 0.22	0.2 ± 0.07	0.08 ± 0.03
E20	33.96 ± 2.73	1.588 ± 0.468	0.21 ± 0.11	1.14 ± 0.24	0.2 ± 0.07	0.07 ± 0.026

#### CAM components

The proportions of the coarse CAM components and their absolute volumes (Fig 7) were estimated by point counting methods. The mesoderm remained the largest component during the entire growth period at about 70%, declining slightly to 60% at E18 but then rising steadily to 75% by the time of hatching. The volume of the chorion was between 10–18% of the CAM volume, being highest at E18 and decreasing to about 10% at E20. The proportion of the allantois remained at around 10%, peaked at E18 to above this value and then declined to about 8% by E20. The proportion of the large vessels appeared to remain steady at about 4–5% of the CAM throughout the incubation period. Notably the proportions of the CAM components did not differ significantly in the various time points during incubation.

The volumes of the components (Table 2) followed closely the trend of the proportions. The highest value for the mesoderm was at E15 while the chorion and allantois peaked at E18. Volumes of all components were decreasing after E18 (Fig 8). The volumes of the CAM components were significantly different at the various time points during incubation. The volume of chorion at E8 was significantly lower than all the other time points except E11. At E11 it was lower than that at E15 and E18. Similarly, chorionic volume was significantly lower at E13 and E20 than at E18. Allantoic volume at E15 and E18 was significantly higher than at both E8 and E11 while mesenchymal volume remained significantly higher in the ages E13-E20 than at E8 and E11. Large vessels at E15 and E18 had significantly greater volumes than at E8 and E11.

The chorion was further analyzed separately by estimating the proportion of the vascular (capillaries) and the non-vascular (chorionic cells) components (Fig 9). The capillaries here were taken to be the endothelia and the lumens of all the capillaries within the chorion. All other cells were considered to be chorionic cells. Between E8 and E11, the chorionic capillaries occupied over 50% of the chorionic volume, but were overtaken by chorionic cells as from E13. By the end of incubation, the capillaries took only about 25% of the chorionic volume. In absolute terms, the values of both the capillary and non-capillary components reached a peak at E18 and then began to decline. The volume of the capillaries remained the greater component up to E11 (Fig 9). Volumes of the chorionic capillaries were significantly greater at E15 and E18 than at E8. Chorionic cell volumes were significantly greater (P≤ 0.05) at all subsequent time points than at E8 and were greater also than those at E11 except at E13. The chorionic cell volume was significantly greater at E18 than at E13.

#### Growth rates

Growth rates of the CAM and its various coarse components were studied by allometry and regression analysis (Fig 9). The CAM volume was seen to increase fastest between E8-E13 (V = 0.22 + M^0.19^), steadily between E13-E18 (V = 1.33 + M^0.03^), and then declined between E18-E20 (V = 3.33 + M^-0.05^), marking three growth phases, identified here as I, II and III respectively. The latter was thus a phase of regression. Body mass-standardized CAM volume decreased gradually through the entire period, and was lowest at E20 (Fig 6). When viewed on individual basis at various growth phases, all components were growing fastest in phase I and, except for the mesoderm, they were regressing in phase III (Fig 9). The chorionic capillary volume grew at similar rates during phase I (V = 0.004 + M^0.003^) and phase II (V = 0.036 + M^0.003)^ but was regressing in phase III (V = 0.25 + M^-0.006^).

In contrast, the volume of chorionic cells was increasing fastest in Phase I (V = 0.001 + M^0.02^), was moderate in phase II (V = -0.005 + M^0.007^) but was declining in phase III (V = -0.46 + M^-0.007^).

#### Expression levels of angiogenesis-related molecules

Relative expression levels (fold change) of selected angiogenesis-related molecules during the course of CAM development were analyzed by real time polymerase chain reaction (RT-PCR (Fig 10). Vascular endothelial growth factor receptor 2 (VEGFR2), the vessel endothelial marker, reached a peak at E11, then decreases steadily, being least at E20. On the contrary, vascular endothelial growth factor (VEGFA) had two peaks one at E11 and again at E20, and was lowest at E8 and E15. The expression levels of hypoxia inducible factor (HIF-1α) followed a pattern similar to that of VEGFA, with peaks at E11 and E20 and were lowest at E8 and E15. Expression levels for the stromal derived factor-1 (SDF-1) had a peak at E11 and then declined steadily towards E15.

## Discussion

Development of CAM in the chick embryo extends between E5 when fusion of the mesodermal layers of the chorion and allantois begins, to E21 when the chick hatches out. The process may conveniently be divided into two parts, the fusion stage and the post-fusion stage. The fusion stage extends from E5 in the chicken embryo when apposition of the chorion and the allantois begins, up to E12 when this process is complete [13] and the CAM covers the entire surface of the egg [15]. In the post-fusion period, the various cellular components of the chorion differentiate, chorionic capillaries assume a superficial position and the allantois increases its strata up to 4. The accelerated differentiation is concomitant with augmentation of sizes for all layers, until E18, when a maximum is reached.

In the current study, we have further divided the developmental stages into three phases based on allometric and regression analysis. The first one extending from E8-E13 is the phase of proliferation and rapid CAM growth. Phase II extends from E13 to E18 and this is the stage of differentiation, growth and expansion and is characterized by rapid expansion of all components to a maximum at E18. Phase 3 extends from E18–E21 and this is the stage of regression and degeneration. Previously, only CAM angiogenesis has been divided into growth phases extending from E5 to E14, based on the presence of sprouts and the sizes of intercapillary tissue meshes, [17, 34], which indicate various stages of angiogenesis [30].

Several studies have previously investigated the developing CAM and the establishment of its tripartite structure and the cellular composition of the various layers is well documented [12]. Proliferation of the endothelial cells was reported to extend up to E11 and by E12 endothelial nuclei were shown to protrude into the capillary lumen[35] [36]. Earlier reports noted that after E14, a few degenerate cells occurred in the chorion and the allantoic cells showed hydrophobic degeneration towards hatching [15]. For the current study, embryo growth phases were determined on the basis of the changing body mass and CAM volume.

The CAM grows in phases reaching a peak at E18 and then starts degenerating through apoptosis. The main changes in the CAM epithelia are reported to occur by incubation days 10 to 11 where striking cellular proliferation becomes a prominent feature in the chorionic layer [12]. However, using PCNA assay we noted sustained cell proliferation up to E14 and by E17, only occasional proliferative cells were encountered. Previously, filopodia containing cells were reported to occur close to the chorionic epithelium at E12 [37] but they were not recognized as participants in the growth of the chorionic epithelium. Filopodia observed here were most prominent in the allantoic cells and emanated from both the epithelial cells and the cells aggregating in the suballantoic layer. The filopodia from the mesenchymal cells established contact with those of the epithelial cells or with the cell walls of the epithelial cells and in so doing became part of the allantois. A similar process occurred in the chorionic layer, an indication that both layers grew by recruitment of subepithelial mesenchymal cells. This observation was further fortified by the fact that in the period of robust growth for the two layers (E11-E14), there was remarkable proliferation of cells in subchorionic and suballantoic parts of the mesenchyme.

Mesenchymal to epithelial transition (MET) has previously been demonstrated in the developing kidney, where advancing nephrogenic tubules recruit cells from the mesenchyme [38]. This transformation of mesenchymal cells was shown to be mediated by Wnt-4 since mice lacking *Wnt-4* activity fail to form pretubular cell aggregates during nephrogenesis [39]. The known molecular regulators of MET in the kidney have been reviewed recently [40]. However, no data exist on the molecular control of the growth of CAM layers. Nevertheless, it was demonstrated that differentiating chorionic cells at E12 CAM were distinguishable through annexin (anx) expression. Annexins are a multigene family of Ca(2+)-regulated phospholipid-binding and membrane-binding proteins that function to control membrane structure and certain membrane transport phenomena [41]. Anx-1 was found in CC and VC cells while anx-2 was localized in capillaries of the chorionic epithelium and basal cells of the allantoic epithelium [37]. Anx-6 occurred in basal cells or endothelial-like cells of the chorionic epithelium and in the media of larger vessels in the mesenchyme [37]. A detailed study of the molecular control of the differentiating CAM layers would further illuminate the preponderant processes.

Morphometric studies on CAM are few and those that are available report either model-based estimates or are conducted on selected few incubation days. In a detailed study, Fitze-Gschwind investigated the chick developing CAM between E5-E21 and noted that the chorionic epithelium increased in thickness from 3.8 ± 1.2 μm at E8 to a maximum of 13.7± 2.2 μm at E19 and then decreased to 9.4 ± 1.5 μm at E20[42]. Elsewhere, Reizis and colleagues reported that the thickness of CAM decreases from 67.4 ± 26.4 μm at E10 to 49.4 ± 20 μm at E14 and then increased steadily to 110.4 ± 38.3 μm at E20 [19]. Later, Maksimov and others indicated that thickness of CAM increases from 61.9 ± 2.48 μm at E7 to a maximum of 82.5 ± 4.95 μm at E10 and then decreased steadily to 41.8 ± 3.17 μm at E20. A stereologic study of the CAM at E16 documented an arithmetic mean thickness of 0.47 μm for the air-blood barrier and a CAM surface area of 90.2 cm^2^ [43].Earlier the arithmetic mean thickness of the air-blood barrier was estimated to be 0.7 ± 0.5 μm and the surface area had been reported to be 63.5 ± 2.5at E20 [42]. The discrepancies in the data reported for the CAM emanate partly due to use of model-based methods, and perhaps also the methods of sampling employed. The decrease of CAM thickness on E14 in the study of Reizis et al was not explained but Maksimov and colleagues note that on E18 after internal pipping, the thickness of the CAM decreases primarily at the expense of the mesodermal layer [18]. This is plausible and is corroborated by the data in the current study. After internal pipping, the lungs become ventilated and the functional blood circulation in the lung is initiated at the expense of the CAM circulation. This results in decrease in blood volume in the vessels of CAM and plausibly this is replaced by water loss from the mesenchyme into the blood vessels. Blood flow through the CAM during incubation continues to increase from 1.3 ml/min at E10 to 4.5–5 ml/min at E18, with a temporal trajectory very similar to that of the egg V̇O2[44, 45] but decreases after internal pipping, when the lung becomes functional. Measurements on in vitro CAM preparations revealed the same developmental pattern and similar blood flow values (Van Golde et al., 1996).

It was previously reported that volume of the chorion increased from 0.012 cm^3^ at E8 to 0.087 cm^3^ at E19 declining to 0.06 cm^3^ at E20 [42]. In the current study the chorionic volume increased from 0.07 ± 0.02 cm^3^ at E8 to 0.37 ± 0.08 cm^3^ at E18 declining to 0.21 ± 0.11 cm^3^ at E20. Notably the latter author used model-based stereology to estimate chorionic volume hence the lower values obtained. Furthermore, in the latter study, chorionic growth was seen to proceed until E19, and then volumes started to decline. In our case CAM and most CAM components continued to grow until E18 then started to regress, except mesenchyme, which peaked at E15. The latter study did not examine data for E18 and hence the highest values captured were at E19.

While the onset of CAM regression may be related to internal pipping, it appears to be a well-programmed process since apoptosis starts well before E18. Local hypoxia ensues in the CAM after internal pipping as indicated by increase in HIF-1α at E18, as blood is diverted to the CAMs[45]. HIF-1α is known to induce transcription of VEGF [46] hence the similar pattern of expression for the two factors. The high expression levels of the three factors (HIF-1α, VEGF and VEGFR2), at E11 is consonant with expectations for a period of robust angiogenesis and consistent with previous observations [21, 22]. The high levels of SDF-1 are a possible indicator for participation of bone-marrow derived mononuclear cells (BMDC) in CAM angiogenesis at E11. Indeed we recently demonstrated that SDF-1/CXCL12 were up-regulated subsequent to inhibition of Notch signaling with a resultant recruitment of BMDC that led to massive intussusceptive angiogenesis [47].

In conclusion, we categorize CAM growth into three phases based on observations from this study. Stage I extends from E4 or 5 to E12 and is the stage of initiation and fabrication of CAM when fusion of the mesodermal components of the chorion and allantois occurs, thus forming the tripartite structure. Stage II extends from E12-E18 and this is the stage of differentiation and expansion. During this stage, various cell types in the allantois and chorion differentiate, the allantois increases in number of layers and the chorion becomes thicker and more complex in structure. Specialized cells such as the calcium transporting VC cells differentiate and gas exchange capillaries become superficially positioned in the chorion. Phase III extends from E18-E21 and this generally is the stage of regression when most CAM components are degenerating. It appears to start after the lung becomes functional and part of the blood is diverted to pulmonary circulation. Nevertheless, degeneration of the CAM is a well programmed process since progressive apoptosis of cells in all layers is evident well before E18.

## Supporting Information

S1 Fig Real-time PCR amplification plot for genes analyzed, showing the log of the change in the fluorescence plotted versus cycle number (ΔRn vs Cycle). The horizontal green line indicates the threshold value of fluorescence.
(PPTX)
