# Supplementary material for: Dynamics of the Developing Chick Chorioallantoic Membrane Assessed by Stereology, Allometry, Immunohistochemistry and Molecular Analysis
Source: PLoS One. 2016 Apr 5;11(4):e0152821. doi: 10.1371/journal.pone.0152821 (PMC4821564; doi:10.1371/journal.pone.0152821)
Supplement: S1 Fig — The horizontal green line indicates the threshold value of fluorescence. (PPTX) [file pone.0152821.s001.pptx]

## Slide 1
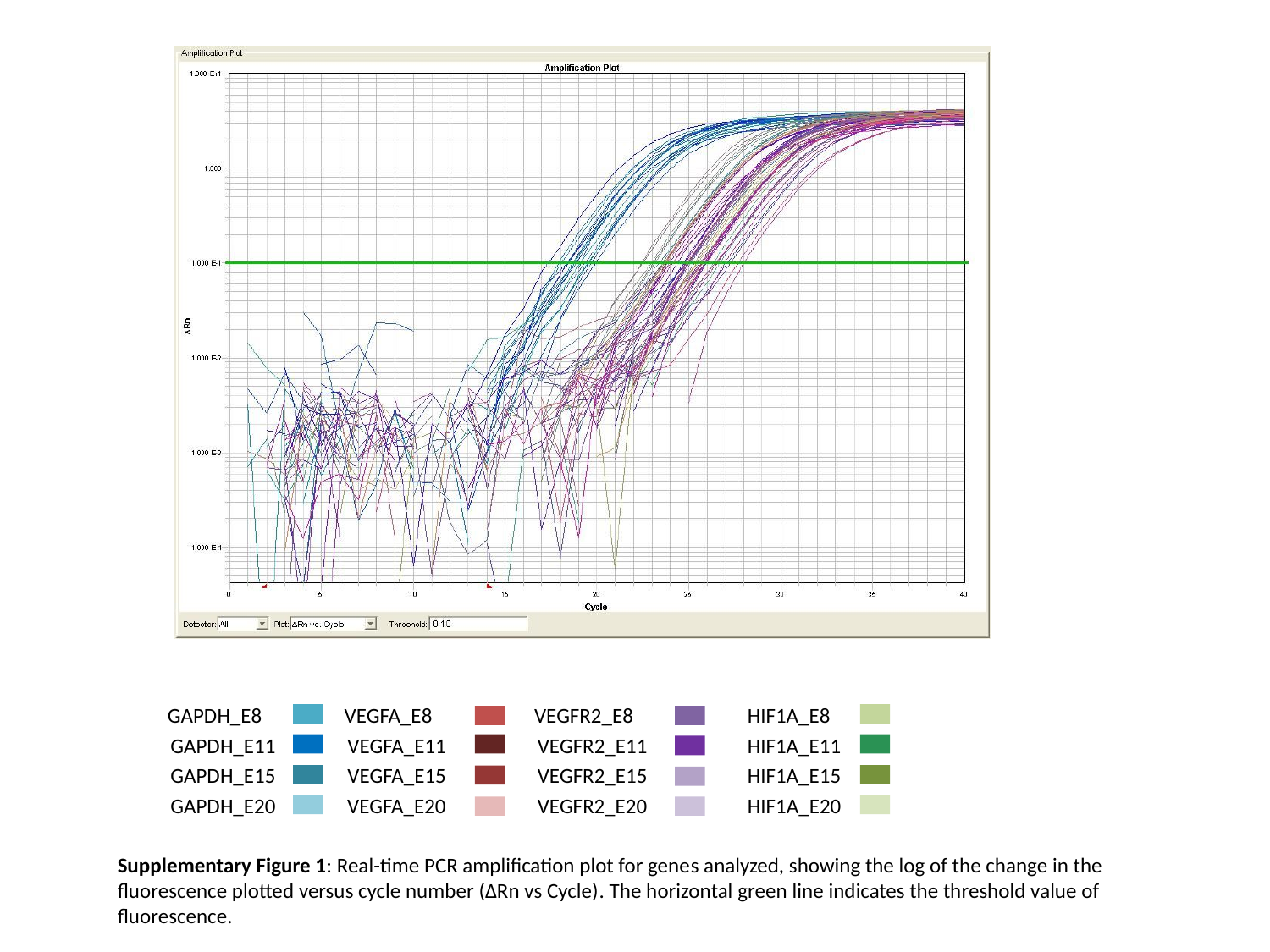

GAPDH_E8
VEGFA_E8
VEGFR2_E8
HIF1A_E8
GAPDH_E11
VEGFA_E11
VEGFR2_E11
HIF1A_E11
GAPDH_E15
VEGFA_E15
VEGFR2_E15
HIF1A_E15
GAPDH_E20
VEGFA_E20
VEGFR2_E20
HIF1A_E20
Supplementary Figure 1: Real-time PCR amplification plot for genes analyzed, showing the log of the change in the fluorescence plotted versus cycle number (ΔRn vs Cycle). The horizon­tal green line indicates the threshold value of fluorescence.
